# Comparison of Two Percutaneous Atrial Septal Defect Occluders for Device Healing and Nickel Release in a Chronic Porcine Model

**DOI:** 10.1155/2020/8413831

**Published:** 2020-06-20

**Authors:** Zakaria Jalal, Younes Boudjemline, Xavier Iriart, Pierre-Emmanuel Seguela, Samantha Delmond, Virgine Loyer, David Gonthier, Olivier Bernus, Marlène Durand, Laurence Bordenave, Jean-Benoit Thambo

**Affiliations:** ^1^Department of Paediatric and Adult Congenital Cardiology, Bordeaux University Hospital (CHU), Pessac F-33600, Bordeaux, France; ^2^IHU Liryc, Electrophysiology and Heart Modeling Institute, Fondation Bordeaux Université, Pessac F-33600, Bordeaux, France; ^3^Centre de Recherche Cardio-Thoracique de Bordeaux, Université de Bordeaux, U1045, Bordeaux F-33000, France; ^4^INSERM, Centre de Recherche Cardio-Thoracique de Bordeaux, U1045, Bordeaux F-33000, France; ^5^Cardiac Catheterization Laboratories, Sidra Cardiac Program, Sidra Medical & Research Center, Doha, Qatar; ^6^CHU de Bordeaux, CIC 1401, Bordeaux F-33000, France; ^7^INSERM, Bioingénierie Tissulaire, U1026, Bordeaux F-33000, France

## Abstract

**Aims:**

To investigate the healing process and nickel release of the Hyperion occluder (Comed BV, Netherlands), as compared to the Amplatzer septal occluder (ASO) (St. Jude Medical Inc., St. Paul, MN, USA) in a chronic swine model.

**Background:**

Some long-term complications occurring after percutaneous atrial septal defect (ASD) closure may be partially associated with an inappropriate healing of the device and increased nickel release. There is no direct comparative study of different occluders for healing and nickel release.

**Methods:**

After percutaneous ASD creation, 12 pigs were implanted with 15 mm Hyperion (*n* = 6) and 15 mm ASO (*n* = 6) devices. After 1 month (*n* = 3 for each device) and 3 months (*n* = 3 for each device) of follow-up, device explantation was performed and healing was assessed using histopathological workup. Systemic and tissular nickel release was performed.

**Results:**

Implantation was successful in 100% without complications. Device coverage was observed as early as 1 month after implantation and was almost complete after 3 months. A granulation tissue with a predominantly mononuclear inflammatory reaction was observed in contact with nitinol wires while an inflammatory reaction was seen in contact with textile fibers. We found no statistically significant difference between the 2 devices whether for histological grading scores or systemic nickel release, regardless to follow-up duration.

**Conclusions:**

In this preclinical study, we demonstrated that Amplatzer septal occluder and Hyperion occluder were not significantly different for device healing and nickel release processes.

## 1. Introduction

Transcatheter device occlusion of secundum atrial septal defects (ASD) has become the currently gold-standard treatment strategy for patients with suitable anatomy [1, 2]. The Amplatzer septal occluder (ASO) (St. Jude Medical Inc., St. Paul, MN, USA) has become the leading device worldwide for closing ASDs in the last two decades owing to its novel design, ease of use, and proven sustained efficacy. Long-term follow-up in both pediatric and adult ASD patients have shown favorable outcome with this device [3, 4]. However, although rare, device closure of ASD is also associated with some potentially serious complications including device thrombosis, device related endocarditis, or migraine headache [5, 6]. It has been suggested that those complications may be partially associated with (1) an inappropriate healing of the ASD closure device and (2) an increased nickel hypersensitivity for migraine occurrence [7–10]. Moreover, cases of incomplete endothelialization from 18 months to 7 years after device implantation have been reported with the Amplatzer septal occluder [11, 12].

These observations led industrial companies which develop ASD occluders to pay great attention to the healing process and to direct their development toward novel materials which are supposed to “accelerate” and provide “fast endothelialization” or “minimize nickel release.”

As for any implantable device, large animal models of ASD have been widely used throughout the historical developments of atrial septal defect closure devices, especially to study the biocompatibility of occluders with a focus on healing and neo-endothelialization processes for regulatory (premarket) device approval [13–16]. However, the great majority of those studies were not comparative as only one device was investigated for each experiment. Recently, the Hyperion ASD occluder (Comed BV, Netherlands) has been developed and commercialized [17]. Its design is identical to that of the ASO. According to manufacturer's product information, this device has 72 preoxidized nitinol wires which are supposed to provide “high biocompatibility,” “fast endothelialization,” and “minimized nickel release.”

The ASO has been historically manufactured using Black Oxide nitinol wire and, since 2014, an additional chemical etching with intaglio has been added. According to manufacturer's product information, this treatment has also been shown to decrease nickel release *in vitro*. However, these properties have not been investigated in *in vivo* preclinical studies so far. Thus, in this study, we aimed to investigate the healing process and nickel ion release of the Hyperion septal occluder as compared to the ASO—used as a gold standard—in a chronic swine model.

## 2. Methods

### 2.1. Technical Specifications of the Devices

We perform our tests on 2 types of devices: one recently developed occluder, the Hyperion occluder, and the ASO which was used as the «gold-standard» device. The Hyperion occluder is very similar to the ASO in terms of design. Each device is composed of a self-expandable nitinol wire mesh with double discs; both discs are attached to each other with a short connecting waist. To increase the occlusive capacity and to ensure a rapid endothelial development according to manufacturer's product information, the 2 discs and the waist are filled with polyethylene terephthalate (device 1) or another polyester (device 2). The technical features of the devices are displayed in Table 1.

### 2.2. Animal Model

All experiments were in line with the European Union Council Directive 2010/63/EU for the protection of animals used for scientific purposes and with local ethical committee approval.

A swine model was used because of the well-developed fossa ovalis and comparable atrial septal anatomy to that of humans. Atrial septal defects were percutaneously created in 12 piglets (median age 82 days (range 76–96); median weight 28 kg (range 26–33)). Animals were premedicated with ketamine (10 mg/kg, intramuscular, Vibrac) and acepromazine (0.1 mg/kg, Vetoquinol). Anesthesia was induced with sodium pentobarbital (5 mg/kg, intravenous, Ceva) and maintained with isoflurane (2% in 100% O_2_, Vibrac).

Septal defects were created by transseptal puncture followed by dilation of the septal defect using a 16 × 30 mm Tyshak Balloon (NuMed, NY, USA), inflated at 2 atm. The diameter of the ASD was then measured by intracardiac echography (ICE). A 0.035-inch guidewire was then placed in the left upper pulmonary vein, and then a 9-Fr delivery sheath was advanced along the guidewire into the left atrium. Subsequently, an occluder 2 mm larger than the created ASD was selected (ASO *n* = 6, Hyperion *n* = 6) and was implanted as previously described [14, 15, 18]. After careful ICE assessment of good device positioning and complete defect closure, the device was released. Animals received heparin at a dose of 100 IU/kg of body weight during the procedure. Before device implantation, 1 g of cefazolin was administered intravenously.

### 2.3. Follow-Up

Animals received aspirin 100 mg/day 1 day prior to the procedure and daily thereafter until termination. For each device, animals were followed up for 1 month (*n* = 6) and 3 months (*n* = 6) after defect closure. Before sacrifice, each animal received an intravenous dose of heparin (400 U/kg body weight) to prevent postmortem clot formation on the device. Euthanasia was performed using sodium pentobarbital (intravenous, 10 mL from 200 mg/mL stock) and the heart was rapidly excised.

### 2.4. Histopathology Evaluation

Immediately after explant, the tissue block containing the implant was dissected free and macroscopic evaluation was performed. The device and the surrounding septal wall were fixed with 10% neutral buffered formalin, included in methacrylate resin, and then cut with a diamond saw (sample thickness 20 *μ*m). The samples were then stained with Richardson's Blue.

Samples from 3 representative locations from the right and left atrial wall including the device were taken and processed for histology. To accurately assess healing process, each slide was scored for inflammation, granulation tissue, and fibrin/thrombus deposition using a 0 to 4 grading system as previously published [19].

Slides were examined at ×10, ×40, ×100, and ×200 magnifications, with NIKON Eclipse microscope, and analyzed with NIS Elements D (3.12 version) software. Endothelialization was further assessed using scanning electron microscopy (SEM). The pathologist was blinded for the type of device implanted and for follow-up duration.

### 2.5. Nickel Systemic and Tissular Release Evaluation

Systemic release: blood samples were taken 1 day before occluder implantation, 1 day after the procedure, and just before animal sacrifice. The serum nickel concentration was measured in each sample by atomic absorption spectrophotometry.

Tissular release: after heart explant, 2 tissue samples were obtained, including 1 sample from right side of atrial septum in close proximity to the device and 2 control samples taken from right ventricular apex, and analyzed for nickel content using inductively coupled plasma/mass spectrometry.

### 2.6. Statistical Analysis

All results are presented as median (range) or number (percentage) when appropriate. Measurement results from Hyperion and ASO devices were compared using Kruskal–Wallis tests, Fisher tests, or split ANOVA plot when appropriate. A *p* value of <0.05 was considered significant. Statistical analysis was performed by SPSS 17.5 (IBM, Armonk, NY).

## 3. Results

**Figure 1:**
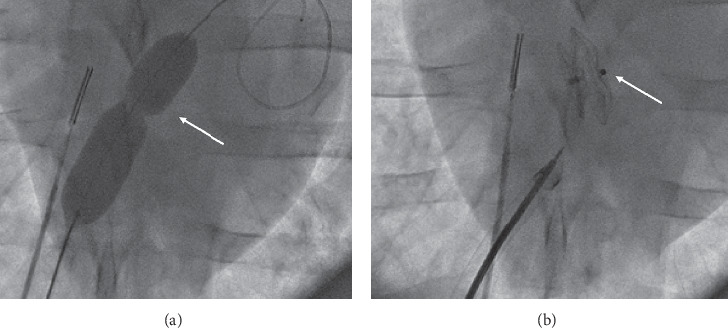
Percutaneous creation of an ASD in a swine model. (a) Fluoroscopic picture of the ASD creation; after transseptal puncture under fluoroscopic and ICE control, a balloon dilatation septum (white arrow) is performed. (b) Fluoroscopic image after ASO release showing satisfactory device positioning (white arrow). ASD: atrial septal defect, ICE: intracardiac echography, and ASO: Amplatzer septal occluder.

Implantation was successful in all animals (100%; *n* = 12/12). The mean procedural and fluoroscopy times were 35 (15–80) and 7.5 (3–28) minutes. Implanted occluder size was 15 mm in all animals. Devices were implanted in appropriate position without residual shunt on echography (Figure 1). Animal sacrifice was performed after a mean delay of 31 (28–38) days (*n* = 6) and 89 (84–91) days (*n* = 6). No relevant clinical complication was observed during the follow-up period.

### 3.1. Macroscopic Assessment

After device explant, no difference was seen between ASO and Hyperion devices on macroscopic assessment. After 1 month of follow-up, all the devices were covered with a thin layer of white tissue. This coverage was almost complete on the right atrial side of the devices (>75% of coverage for all studied devices) and was predominantly observed on device periphery bordering the defect edges. The left atrial side of devices was only partially covered (25% to 50% of coverage for 1 ASO device, 50 to 75% of coverage for the remaining occluders) and no coverage was observed on device screws and hubs.

**Figure 2:**
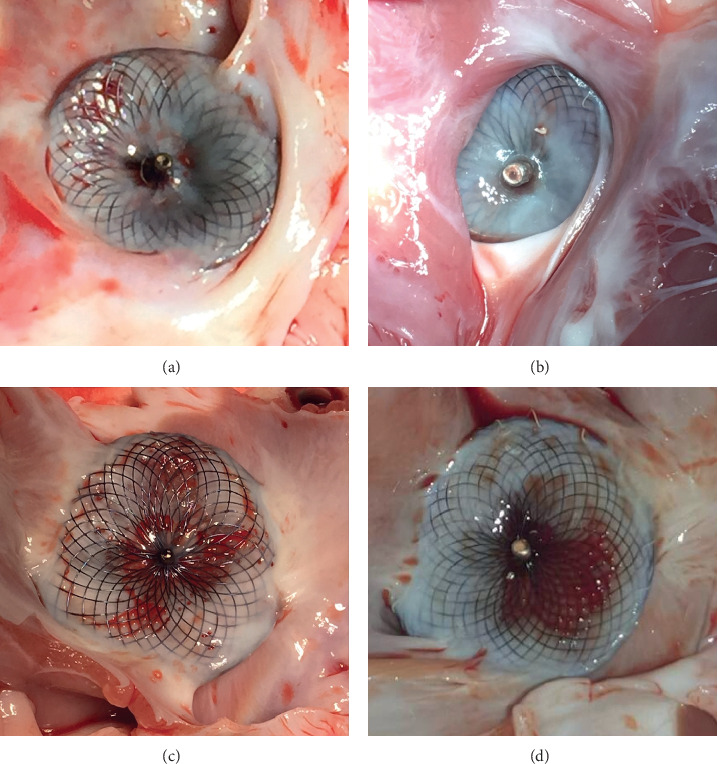
Macroscopic aspects of the 2 occluder devices with different implantation times. Photos of the devices were taken immediately after sacrifice and dissection of the hearts. (a) Superficial device coverage in an ASO 1 month after implantation (right atrial side). (b) Advanced device coverage on the right atrial disk of a Hyperion occluder 3 months after implantation. (c) Aspect of the left atrial disk of an ASO 1 month after implant, the covering tissue is predominantly observed on device periphery bordering the defect edges. (d) Advanced device coverage of a Hyperion occluder 3 months after implant, left atrial disk.

After 3 months of follow-up, devices coverage layer was thicker and almost complete on both right and left device discs (>75% of coverage for all devices). Device screws and hubs were still uncovered for all devices. No deformations or wire strut fractures were observed. No thrombus formation was detected on the surface of the devices (Figure 2).

### 3.2. Histopathology

By 1 month after implantation, a granulation tissue covering from 50% to 100% of device surface was observed. It was associated with a predominantly mononuclear inflammatory reaction (macrophages, lymphocytes) in contact with nitinol wires. We also observed an intense inflammatory reaction with accumulation of multinucleated giant cells in contact with textile fibers. The fibrous connective tissue structure was generally more organized, on the right disc with a fibroblastic organization of surface which was covered by endothelium-like cells. The presence of small fibrin condensations was seen in 4/6 devices, in contact with nitinol wires (*n* = 2) and the device screw (*n* = 2).

**Figure 3:**
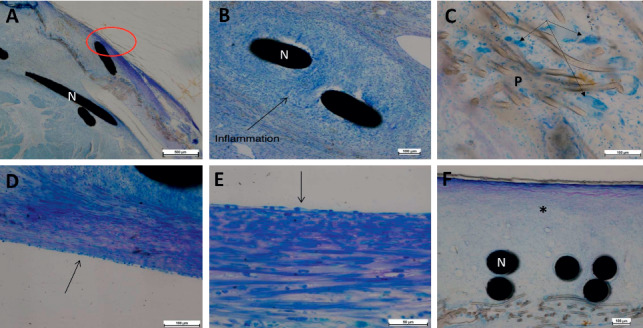
Histological images with Richardson Blue staining showing sequential tissue organization of device coverage after implantation. (A–C) Right atrial disk of an ASO 1 month after implant, showing a thin coverage of the device ((A), red circle) associated with an inflammatory reaction including mononuclear directed toward the nitinol wires (B) and foreign body giant cells (black arrows) in contact with the polyester fibers (C). (D–F) Right atrial disk of a Hyperion occluder 3 months after implant; the granulation tissue is less inflammatory but more fibrous (D, E) with a more organized and vascularized pattern (black asterisk, (F)). We can observe an integration of nitinol wires (F) associated with a superficialneo-endothelial layer (black arrows, (D, E)). ASO: Amplatzer septal occluder; N: nitinol; P: polyester fibers.

At 3 months after implantation, the nature of the observed granulated reaction was histologically different: the tissue was less inflammatory but more fibrous and vascularized, with an integration of nitinol wires. The inflammatory reaction directed toward textile fibers was still the same. No fibrin or thrombotic material was observed except a small condensation observed on the left disc of 1 Hyperion device (Figure 3).

When comparing the grading scores for inflammation, granulation tissue, and fibrin/thrombus deposition of the 2 devices, we found no statistically significant difference (Table 2), whether after 1 month or 3 months of follow-up.

### 3.3. Scanning Electron Microscopy

**Figure 4:**
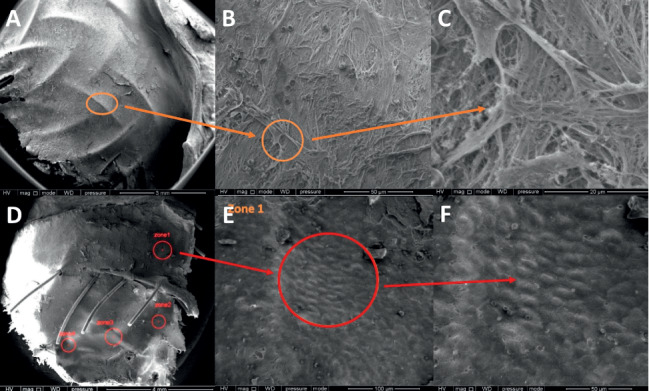
Scanning electron microscopy images showing device coverage. (A)–(C) Right atrial disk of an ASO 3 months after implantation; device covering is complete (A) with large areas of acellular fibrotic tissue (B, C). (D)–(F) Left atrial disk of the same ASO 3 months after implantation, and endothelial cells were observed as soon as 1 month after implantation and were more or less organized in cells clusters with cellular fibrotic tissue displayed in white (E, F). ASO: Amplatzer septal occluder.

SEM performed on different specimens confirmed the results of optical microscopy. Endothelial cells were observed as soon as 1 month after implantation and were more or less organized in cells clusters. Otherwise, endothelial cells had a patchy distribution, leaving some large areas of acellular fibrotic tissue. No difference between ASO and Hyperion devices could be observed on SEM samples (Figure 4).

### 3.4. Nickel Release

#### 3.4.1. Systemic Release

Table 3 displays median serum levels of nickel throughout the study period. We found no significant difference between the 2 studied devices for systemic nickel concentrations (ASO vs Hyperion at 1 month: *p*=0.1; ASO vs Hyperion at 3 months: *p*=0.9). For one studied single device, there was a trend toward a significant increase of nickel levels for ASO after one month (*p*=0.06) compared with baseline but not after 3 months. No difference was observed between baseline and follow-up nickel levels for Hyperion device.

### 3.5. Tissular Release

Median interatrial and right ventricular apex nickel tissular levels were 7 (4.6–17.4) and 7 (5.8–7.6) ng/mL for ASO at 1 month; 5 (4.5–7) and 0 (0–3.6) ng/mL for ASO at 3 months; 5 (4.3–7.1) and 4 (0–4.9) ng/mL for Hyperion at 1 month; 7 (4.8–7.5) and 7 (4.5–15.7) ng/mL for Hyperion at 3 months. No difference was observed between the 2 devices regarding interatrial tissular release whether after 1 month or 3 months of follow-up. Regarding RV apex, tissular nickel levels were significantly higher with ASO devices at 1 month (*p*=0.043), significantly higher with Hyperion devices at 3 months (*p*=0.046).

## 4. Discussion

This experiment series is, to our best knowledge, the first which aimed to evaluate and compare 2 commercially available ASD occlusion devices for healing and nickel release in a large animal model. In this work, we showed that, despite differences in terms of design, material, or coating, the 2 studied occluders, i.e., Amplatzer septal occluder and Hyperion occluder, were not significantly different for healing and systemic or tissular nickel release.

### 4.1. Device Coverage

In this experiment, we found that device coverage was observed as early as 1 month after implantation. Device coverage started on occluder periphery bordering the defect edges and included (1) more or less organized endothelial cells clusters, (2) a granulation tissue with a predominantly inflammatory reaction in contact with nitinol wires, and (3) an intense inflammatory reaction in contact with textile fibers. The coverage was almost complete after 3 months, with a less inflammatory but more fibrous and vascularized tissue integrating nitinol wires whereas the inflammatory reaction directed toward textile fibers was still the same. Regardless of the type of occluder, our results are in line with the great majority of previously published animal experiments [14–16, 18]. In addition, we observed no coverage of the devices screw threads after 3 months of follow-up. This is also consistent with previous reports, in which the protruding parts of the metal framework of the devices were the last parts of the occluders to be covered (usually after 3 to 5 months of follow-up) [18, 20].

Sigler et al. compared the healing of ASO and Starflex devices in 2 series of human and animals' experimental explants [18, 21]. The devices implant ranged from 5 days to 48 months in humans and 4 days to 12 months in sheep ASD model. Early neo-endothelial coverage was observed to be equally distributed over the entire surface of the device without a preference of the device periphery bordering the defect edges. Conversely, we observed that device coverage was predominantly observed on occluder's periphery bordering the defect edges.

Two hypotheses may explain this difference: (1) in their experiments, Sigler and colleagues implanted a great majority of Starflex occluders whose design is completely different from that of ASO or Hyperion; (2) the authors did not mention the size of the implanted devices and the degree of occluders oversizing, which both play a key role for the final device shape, especially in occluders periphery. Nevertheless, after a follow-up period of >90 days, analyzed samples showed a complete neo-endothelial coverage, as shown in our work [14–16, 18, 21].

Industrial companies' claims usually argue the superiority of their device material, design, or coating which are supposed to “accelerate” or provide “fast endothelialization.” Indeed, as some long-term complications following percutaneous ASD closure have been shown to be associated with an inappropriate healing of the device [5, 6], industrial companies pay a great deal of attention on device healing process. As a result, the choice of a new material, coating, or design for device development supposed to provide a faster endothelialization is used as a selling point, based on animal studies which are only descriptive and, to the best of our knowledge, not comparative. From this point of view, despite some inherent limitations, our study has the merit to compare for the first time 2 commercially available devices for both healing and nickel release, and to put in perspective the industrial companies' claims regarding the potential superiority of a device.

### 4.2. Nickel Release

Nitinol alloy (composed of 45% titanium and 55% nickel) is widely used in interventional cardiology field because of its shape memory, good radiopacity, magnetic resonance imaging compatibility, and resistance to fatigue and corrosion. In case of ASD occluders, the high nickel content might be an issue, as concerns have been raised about the potential hypersensitivity reactions due to nickel release [5]. Following a percutaneous closure, the most frequent symptom associated with nickel release and hypersensitivity is migraine headache but chest pain, rash/urticaria, difficulty breathing, fever, or pericardial effusion with tamponade have also been described [22, 23]. A large study of international registries noted nickel allergy as the most common reason for device explantation for patent foramen ovale devices [24].

Therefore, similar to the healing process, industrial companies pay a great deal of attention on nickel release and direct their development toward novel nitinol treatment or coating (such as the use of intaglio for the ASO since 2014). In this series, no significant difference was seen between the 2 devices regarding systemic nickel content, whether after 1 or 3 months of follow-up. Furthermore, serum nickel levels were very low, for both devices.

Comparative study of ASD occluders for nickel release has rarely been performed. Verma et al. compared *in vitro* nickel elution properties of ASO, Gore septal occluder, and Helex septal occluder. Nickel content was measured at 14 intervals over 90 days. They showed that nickel elution was significantly higher for ASO, compared with other devices, as early as 24 hours, and was still elevated at 90 days [25]. Other authors published both *in vitro* and *in vivo* comparison of nickel release between uncoated and titanium-nitrogen- (TiN-) coated occluders [16, 20]. They showed that TiN coating significantly reduced the release of nickel both *in vivo* and *in vitro* indicating an improved biocompatibility of the occluders. However, these latter studies were performed during TiN coating validation process of a new occluder. No comparison between these TiN-coated occluders and a commercially available device was performed.

### 4.3. Study Limitations

Our study has several limitations. First, this work was limited by a small sample size and a short follow-up period of animals. Second, the percutaneous creation of the defect using transseptal puncture and subsequent balloon dilation might lead to a fresh wound in the septal wall that could potentially alter the healing response to devices implanted thereafter. The human heart is known to respond in a different fashion to porcine species for endothelialization process, making our results only partially predictive.

## 5. Conclusions

In this preclinical study, we demonstrated that, despite differences in terms of design, material, or coating, Amplatzer septal occluder and Hyperion occluder were not significantly different for healing and systemic or tissular nickel release. Although having several limitations, this work has the merit to put in perspective the industrial companies' claims regarding the potential superiority of a device in terms of healing, endothelialization, or nickel release.

## Figures and Tables

**Table 1:** Technical features of the occlusion devices.

Type of device	Device 1 Hyperion ASDO	Device 2 Amplatzer
Metal alloy	Nitinol	Nitinol
Wire thickness	150 microns	190 microns
Mesh structure	Polyethylene terephthalate	Polyethylene terephthalate
Nitinol treatment surface coating	Preoxidized nitinol wires	Black oxide nitinol Intaglio coating

ASO: Amplatzer septal occluder; ASD: atrial septal defect; NA: not available.

**Table 2:** Summary of median (range) histopathological scores for ASO and Hyperion devices.

	Inflammation	Granulation tissue	Fibrin/thrombus
ASO 1 month (*n* = 3)	3 (3-4)	3 (1–4)	1 (1-2)
ASO 3 months (*n* = 3)	3 (3-3)	3 (2-3)	0 (0-1)
Hyperion 1 month (*n* = 3)	3 (3-4)	3 (2-3)	1 (0-1)
Hyperion 3 months (*n* = 3)	4 (3-4)	3 (3-4)	1 (0-1)
ASO vs Hyperion 1 month	*p*=0.9	*p*=0.9	*p*=0.9
ASO vs Hyperion 3 months	*p*=0.9	*p*=0.9	*p*=0.4

**Table 3:** Median (range) serum levels of nickel along the study period.

	J0 (ng/mL)	J3 (ng/mL)	Before sacrifice (ng/mL)	*p*
ASO 1 month (*n* = 3)	4 (0–4.5)	4 (3.5–4.7)	5 (5.1–6.3)	0.066
ASO 3 months (*n* = 3)	4 (3.5–5.6)	5 (3.5–6)	5 (4.6–6.7)	0.213
Hyperion 1 month (*n* = 3)	5 (4.8–6.5)	5 (5–5.6)	5 (4.4–5.5)	0.837
Hyperion 3 months (*n* = 3)	3 (0–6.4)	4 (0–5.4)	5 (4.9–5.7)	0.415

## Data Availability

All the data supporting the results and conclusions are included in the article.
